# Atypical Fibroxanthoma Within a Melanoma: A Case Report

**DOI:** 10.7759/cureus.20426

**Published:** 2021-12-15

**Authors:** Dujanah S Bhatti, Dharshanan Raj Sela Raj, Muhammad Adil A Khan, Raheel Ahmad, Nur Ul Ain, Louise J Smith

**Affiliations:** 1 Plastic and Reconstructive Surgery, Aberdeen Royal Infirmary, Aberdeen, GBR; 2 Plastic Surgery, Aberdeen Royal Infirmary, Aberdeen, GBR; 3 Plastics Surgery, Aberdeen Royal Infirmary, Aberdeen, GBR; 4 Surgery, Oxford University Hospitals NHS Foundation Trust, Oxford, GBR; 5 Plastic and Reconstructive Surgery, Holy Family Hospital, Rawalpindi, PAK; 6 Patholgy, Aberdeen Royal Infirmary, Aberdeen, GBR

**Keywords:** plastic and reconstructive surgery, skin lesions, ­skin cancer, melanoma surgery, atypical fibroxanthoma

## Abstract

The finding of a pigmented lesion within another distinct lesion is rare but not unheard of. Here, we describe the presence of an atypical fibroxanthoma within a melanoma in a 72-year-old female referred to the plastics surgery department with a pigmented lesion on her left knee. It was excised in view of clinical suspicion of melanoma. The histopathology report documented a single lesion with two distinct components, namely a melanoma of superficial spreading type with a Breslow thickness of 3.0mm, and a central nodule of atypical fibroxanthoma.

## Introduction

Melanoma and atypical fibroxanthoma (AFX) are two distinct skin tumours that may present as pigmented lesions and can clinically mimic each other. However, less commonly two types of tumours can present simultaneously in one lesion. The lesion is then known as a collision tumour [1]. This case report concerns a very rarely documented collision tumour involving melanoma with an AFX. According to a Medline search, only two cases of this type of lesion have been previously recorded [2]. The literature has highlighted the presence of melanoma with the mimic of atypical AFX; however, the concomitant presease of AFX within the melanoma has little literature.

## Case presentation

A 72-year-old female was referred by her general practitioner to the plastic surgery department with a three-month history of the sudden growth of a longstanding pigmented lesion on the medial aspect of her left knee. She had type I skin and denied any sunbed use or excessive sun exposure. She was an ex-smoker of 20 cigarettes a day who stopped four years ago. Her only significant past medical history consisted of hypertension and depression.

Examination revealed a 2 x 1cm raised, nodular, pigmented lesion in the medial aspect of her left knee, for which an excision biopsy was performed due to suspicion of melanoma. Macroscopically, the nodule was well-circumscribed with partly pale cream and partly light grey surface, 12 x 10 x 8mm. There was an adjacent light brown plaque, 7 x 6mm, in continuity with the nodule.

**Figure 1:**
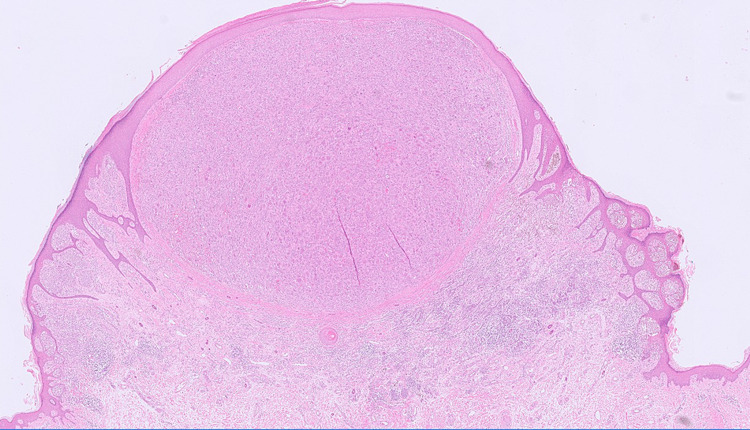
Whole view of melanoma and AFX (H&E) showing an overall nodular lesion with two distinct components. There is a central dermal nodule of AFX with a surrounding invasive melanoma component. AFX - atypical fibroxanthoma

**Figure 2:**
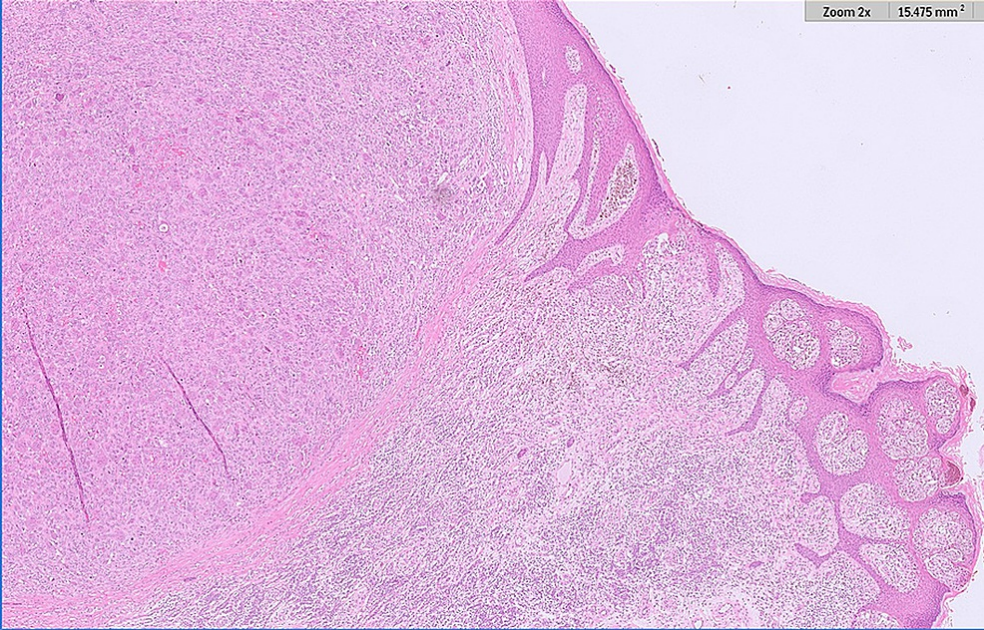
Higher power view illustrating the invasive melanoma component (to the right of the image) and the AFX component (to the left of the image) (H&E). AFX - atypical fibroxanthoma

**Figure 3:**
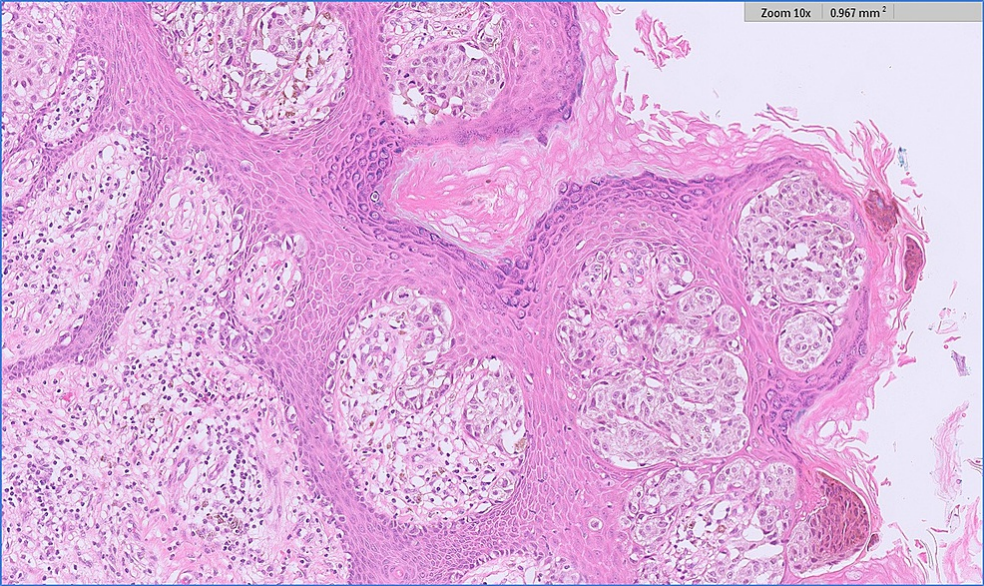
High power view of melanoma mainly showing its junctional component with enlarged, atypical, epithelioid melanocytes. The melanocytes are mainly forming nests and some of these are ascending through the epidermis (H&E).

**Figure 4:**
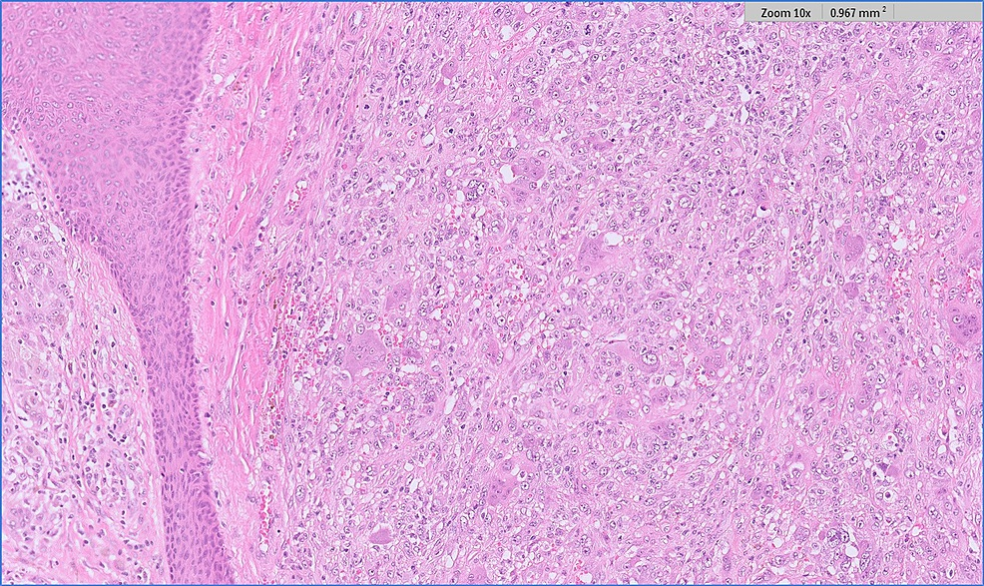
High power view of AFX component composed of enlarged, atypical, and mononuclear cells with vesicular nuclei and frequent mitoses, including atypical mitoses. The mononuclear cells are admixed with many atypical multinucleated giant cells (H&E). AFX - atypical fibroxanthoma

Microscopic examination showed a single epidermal and dermal-based lesion with two distinct histological components therein (Figures 1, 2). At either side of the overall lesion, there was an invasive malignant melanoma of the superficial spreading subtype (Figure 3). The melanoma had a Breslow thickness of 3.0mm with no evidence of ulceration. The pathological stage for melanoma was pT3a (AJCC 8) and clinical stage 2A. At the centre of the melanoma, there was also a well-circumscribed but unencapsulated dermal nodule with an elevation of the overlying epidermis. This distinct nodule was composed of many osteoclast-like multinucleated giant cells set in a background of numerous atypical mononuclear cells with marked nuclear pleomorphism and a high mitotic count, including atypical mitoses (Figure 4). The central nodule was confined to the dermis and had a well-defined, regular border. Within the central nodule, there was no evidence of necrosis, no vascular space invasion, and no perineural infiltration.

**Figure 5:**
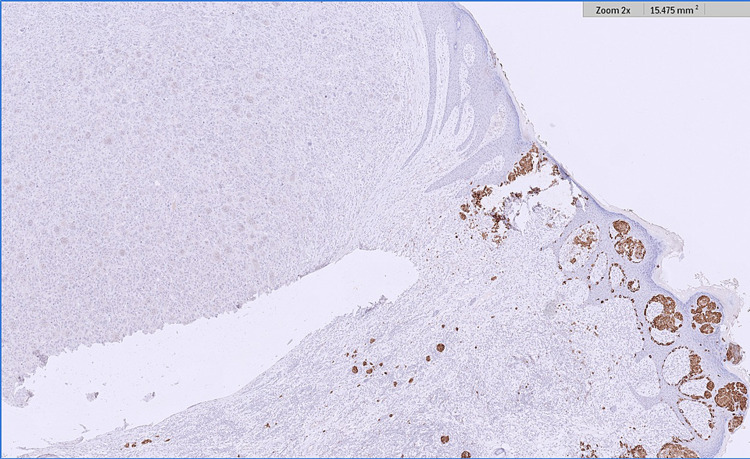
Melan A immunohistochemistry illustrating positive staining in the invasive melanoma component (to the right of the image) and negative staining in the AFX component (to the left of the image). AFX - atypical fibroxanthoma

**Figure 6:**
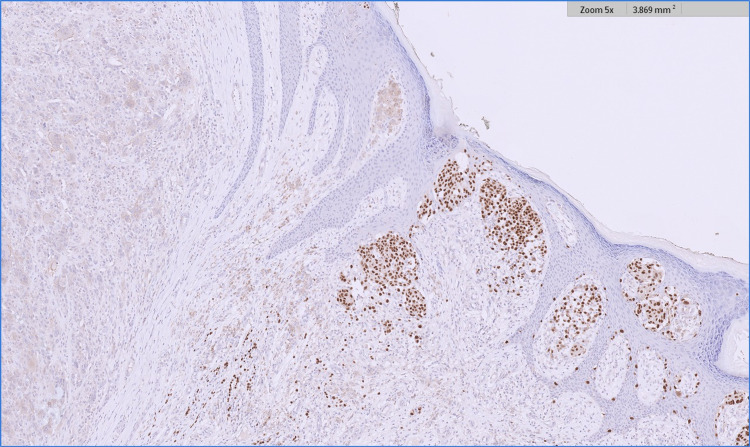
SOX-10 immunohistochemistry illustrating positive staining in the invasive melanoma component (to the right of the image) and negative staining in the AFX component (to the left of the image). AFX - atypical fibroxanthoma

**Figure 7:**
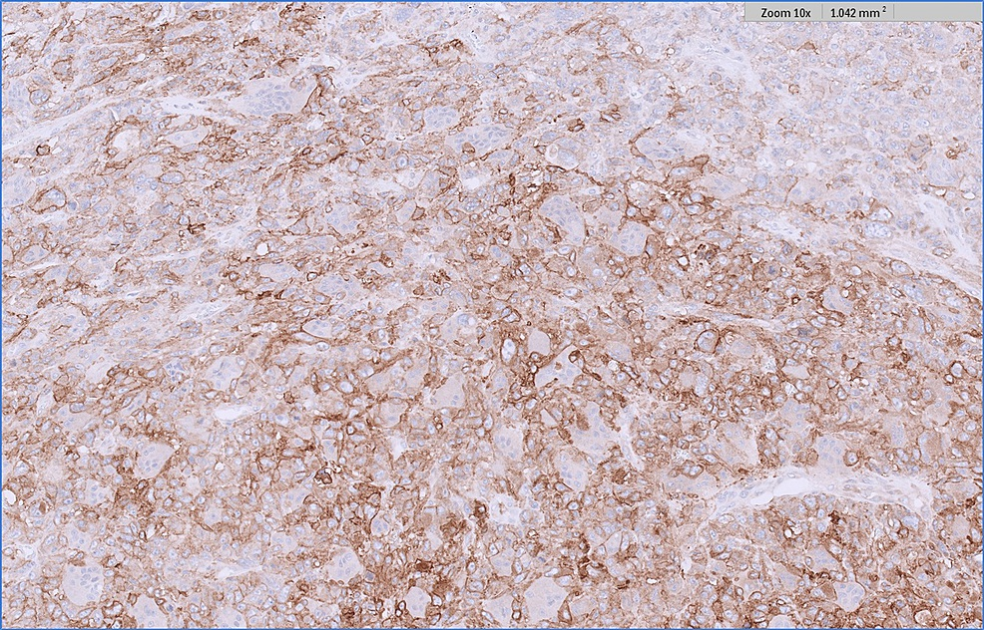
CD 10 immunohistochemistry showing strong positive staining within the AFX component. The melanoma component proved negative (not shown).

**Figure 8:**
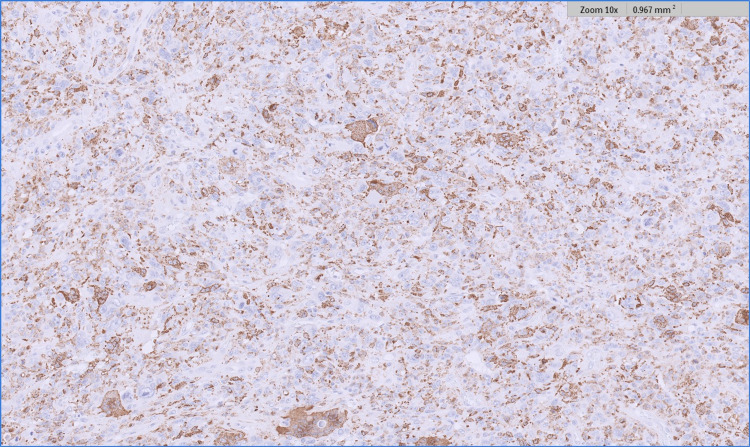
CD 68 immunohistochemistry (a macrophage marker) showing positivity in the multinucleated cells of the AFX component. This marker was negative in the melanoma (not shown). AFX - atypical fibroxanthoma

**Figure 9:**
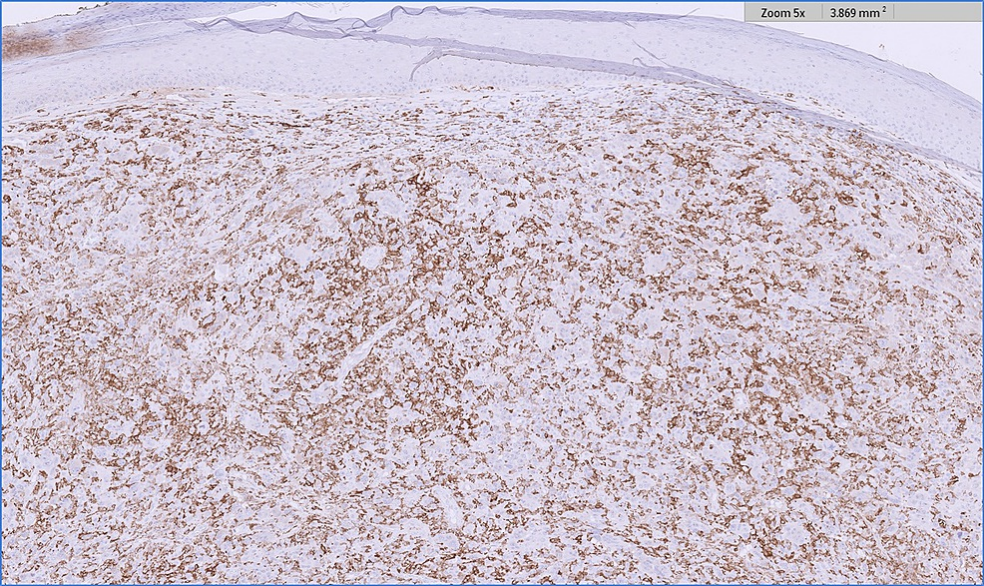
CD 163 immunohistochemistry (a macrophage marker) showing positivity in the mononulcear cells of the AFX component.  This marker was negative in the melanoma (not shown). AFX - atypical fibroxanthoma

On immunohistochemistry, the melanoma component showed Melan A and SOX-10 positivity[LS(G1], whilst these markers were negative in the central nodule (Figures 5, 6). The discrete, central nodule showed positive staining with CD 10, CD 68, and CD 163, whilst all of these markers were negative in the melanoma component (Figures 7-9). Ki-67 showed a high proliferation index in the central nodule. S100, HMB-45, cytokeratin MNF 116, high molecular weight cytokeratin, p63, and smooth muscle actin were all negative in the central nodule.

Morphologically and immunohistochemically, the central nodule was distinct from the surrounding melanoma. The central nodule was diagnosed as AFX. Both the central nodule and the surrounding invasive malignant melanoma were well excised.

BRAF mutation analysis was carried out on the melanoma component. No mutation was detected at codon 600 of the BRAF gene using direct sequencing analysis. Subsequently, wide local excision of the surgical scar and sentinel lymph node biopsy of two nodes from the left groin was undertaken. The wide local excision showed scarring only. Both sentinel lymph nodes were benign.

## Discussion

Both melanoma and especially AFX can be varied in their clinical presentation and, as a result, have a myriad of differential diagnoses. They have also been known to present as clinical mimics of each other. Correspondingly they require clinical, histological, and, particularly in the case of AFX, immunohistochemical evaluation for accurate diagnosis. This can represent a challenge for clinicians, yet it is vitally important given their differing clinical courses and prognoses. Exceedingly rarely, however, these skin lesions can occur together, as is demonstrated in this case report. In a typical, straightforward melanoma, immunohistochemistry is not necessary for diagnostic purposes, however, we utilised it here to further demonstrate that the central nodule of AFX was a completely different lesion to the surrounding melanoma in having distinct morphology and immunohistochemical features.

AFX was first described in the 1960s by Helwig and was believed to be of fibrohistiocystic origin [3]. Most lesions present as rapidly growing, ulcerated or bleeding, red nodules or plaques on sun-exposed areas [4]. They are relatively rare, accounting for only 0.2% of skin cancers [5]. Histologically, they represent a low-grade superficial type of cutaneous sarcoma [4].

There appear to be two distinct patterns in the development and course of AFX lesions. The first pattern of lesion commonly occurs in the sun-exposed skin of elderly patients (scalp, face, ears, and upper limbs). The second group develops out-with sun-exposed areas and tends to affect younger patients, including children, with underlying conditions that result in defective DNA repair, such as xeroderma pigmentosum [6]. These lesions are often larger but slower growing. The case reports of McGregor and Wilsher concerning the collision of melanoma and AFX similarly described lesions in elderly patients, although both were male and in the more typical sun-exposed regions of the head and neck [1,2]. Contrastingly, in this case report, the lesion was in a far less common site on the lower limb.

Ultraviolet radiation from sunlight is an important risk factor in the development of many skin cancers, including AFX and melanoma [6]. Radiation causes damage to the DNA repair mechanisms, including the p53 tumour suppression protein. Other risk factors include previous radiotherapy and immunosuppression, in particular for recipients of organ transplants [4].

The list of clinical differential diagnoses for AFX and melanoma are varied, and they are also differentials for each other. These differentials include squamous cell carcinoma, basal cell carcinoma, Merkel cell carcinoma, dermatofibrosarcoma protuberans, malignant fibrous histiocytoma (MFH), and malignant schwannoma [3]. Very rarely, cases of amelanotic melanoma mimicking AFX lesions have been documented [7].

AFX lesions are usually pink to red in colour, but maybe pigmented, and are normally less than 2 cm in diameter [6]. Clinical examination with the aid of a dermatoscope typically reveals a lesion with polymorphic vessels radiating to the centre with surrounding white areas [4,6]. The histology of a typical AFX is an often polypoid, dermal-based, frequently ulcerated lesion that is reasonably well-defined. Cytologically, AFX can vary widely from spindle-cell predominant variants or polymorphic lesions with bizarre cells [8]. Nuclei are hyperchromatic and multilobated with numerous mitotic figures [8]. Some lesions include cells with lipid-containing cytoplasm resembling a xanthoma and hence where AFX derives its name.

Given the similar clinical presentation and appearance of melanoma, AFX, and other skin lesions, immunohistochemistry forms an important part of the diagnostic process. Malignant melanomas are typically positive for S-100 protein, Melan-A/MART1, HMB45, and SOX-10 (although expression of these markers can vary according to how well-differentiated the melanoma is), while these markers are not usually seen in AFX [6]. CD68 and CD163 (macrophage markers) can be expressed in AFX [4] and were strongly positive in this case. Cytokeratin expression is found in squamous cell carcinoma but is rare in AFX [6]. CD10 is more typically positive in AFX [6] as was the case here. Immunohistochemistry was essential in this case to diagnose the nodular (AFX) part of the lesion, specifically in determining that it was not melanoma or squamous cell carcinoma. The lesion in the Wilsher (2009) [1], a case report was positive for CD99, CD10, CD68, S-100, Melan A, and HMB45.

AFX has a favourable prognosis and is usually curable by complete surgical excision or Mohs microsurgery. Rarely, it can recur locally or metastasise [4]. AFX-like melanoma carries a poorer prognosis. Typical melanoma can be very aggressive (taking into consideration the Breslow thickness), therefore, requiring prompt identification, treatment, and appropriate follow-up [7].

## Conclusions

Clinically and histologically, melanoma can resemble and mimic other cutaneous lesions. This presents as a challenge for clinicians and pathologists to clearly diagnose and propose treatment options. We reviewed a patient who had a rare finding of an AFX within an invasive melanoma. Both morphology and immunohistochemistry were used to diagnose the two distinct lesional components. Following discussion of this case at the Melanoma Multidisciplinary meeting, a treatment and follow-up plan was put in place (as per local guidelines), with the clinicians having the awareness of the AFX and invasive melanoma components in this 72-year-old female.
